# Controlled, Bio-inspired Self-Assembly of Cellulose-Based Chiral Reflectors

**DOI:** 10.1002/adom.201400112

**Published:** 2014-05-30

**Authors:** Ahu Gumrah Dumanli, Gen Kamita, Jasper Landman, Hanne van der Kooij, Beverley J Glover, Jeremy J Baumberg, Ullrich Steiner, Silvia Vignolini

**Affiliations:** Department of Physics, University of CambridgeJJ Thomson, Cambridge, CB3 0, HE, UK; Department of Chemistry, University of CambridgeLensfield Road, Cambridge, CB2 1EW, UK; Department of Plant Science, University of CambridgeDowning Street, Cambridge, CB2 3EA, UK

**Keywords:** cellulose nanocrystals, self assembly, photonics, chirality, biomimetics, cellulose

Layered transparent photonic stacks are known to give rise to highly brilliant color in a variety of living organisms.[1] The biomimetic replication of these structures not only offers a wide range of applications, but can also be used as a tool to gain understanding of the biological processes responsible for the self-assembly of these structures in nature. Recent studies showed that cellulose microfibrils form helicoidal stacks in the plant cell wall, which selectively reflect circularly-polarised light of a specific wavelength.[2]–[5] Such structures are responsible for the bright colors in fruits[2] and leaves[3] of very different species of plants.[4,5] Similar photonic structures can be artificially produced using the same constituent material, cellulose nano-crystals (CNCs).[6,7] Slow evaporation of a CNC suspension gives rise to their spontaneous assembly into a chiral nematic liquid crystalline phase that can be preserved in the dry state.[8,9] The self-assembly process is strongly dependent on the properties of the nanoscale building blocks and on the macroscopic parameters that characterise the assembly.[10]–[12] Many factors influence the optical and mechanical properties of the obtained film, including temperature and pressure[13,15,16] the substrate,[14] and the surface chemistry of the CNCs.[17,18] Nevertheless the self-assembly process is robust and can be coupled with a range of chemical processes.[19]–[21]

Here, we introduce a technique to control and optically monitor the self-assembly of CNCs from a suspension during the formation of a structurally colored film with chiral nematic morphology. By adjusting the environment during film formation we are able to vary the dynamics of color formation. The optical properties of the fabricated samples were investigated both in terms of their microstructure by scanning electron microscopy and spectroscopically using circularly and linear polarised light microscopy. We find that the loss of water from between and within the CNCs, which is influenced by the humidity, controls the final nanostructure and therefore the film color.

The optical properties of the fabricated CNC films are similar to the fruit of *Pollia condensata* (**Figure 1**). The appearance of the fruit is the result of selective color reflection from a cellulose-based helicoidal structure in the epicarp cell wall.[2] Each cell reflects circularly-polarised light of specific handedness and color[22,23] giving rise to its pointillistic appearance. In particular, the reflected color λ_r_ (for normal incidence) depends on the pitch (*p*) of the helicoid (defined as the distance over which is observed a 180° rotation of the chiral nematic director) and the average refractive index n_av_ of the film, as demonstrated by H. De Vries:[22]

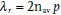
1

**Figure 1:**
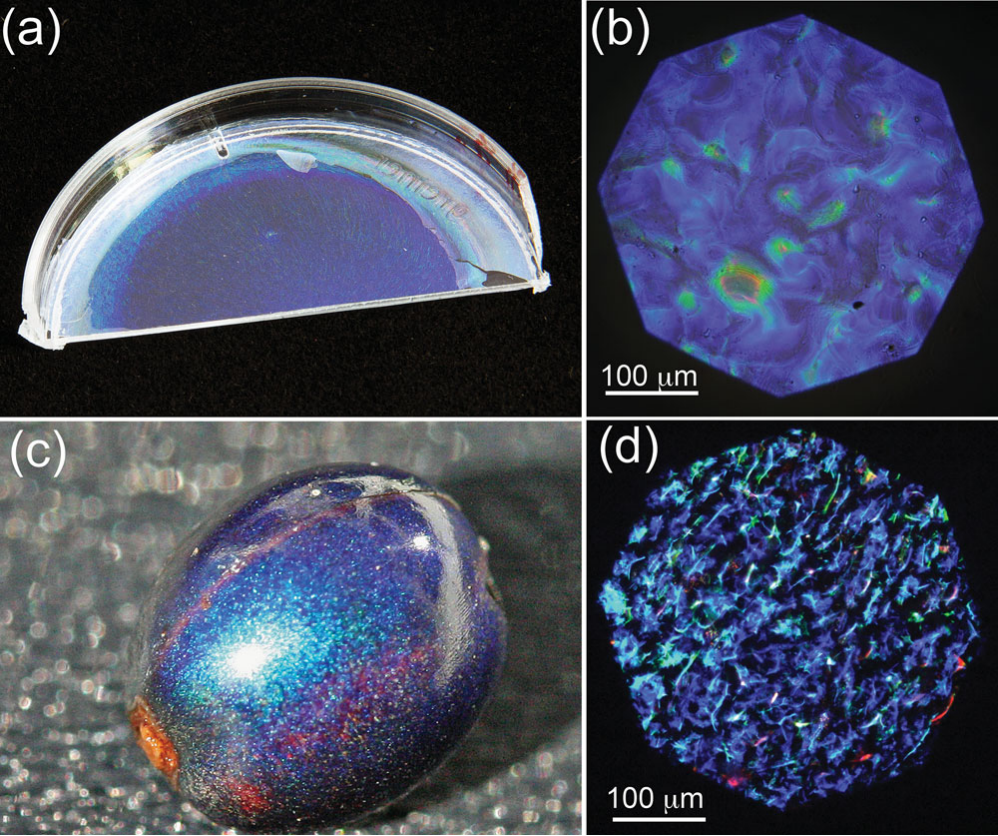
Biomimetic cellulose film compared with the *Pollia condensata* fruit. Panels (a) and (c) are photographs of the biomimetic film and the fruit, respectively. Panels (b) and (d) show optical microscopy images in cross polarisation configuration of the film and the fruit, respectively.

Similarly the fabricated cellulose sample selectively reflects circular-polarised light. The optical microscopy images were collected in cross polarisation configuration using a 20× objective with numerical aperture NA = 0.2.

Cellulose is the most abundant biopolymer on the planet. In nature, cellulose is found mainly in the plant cell wall in various hierarchical fibrillar forms, depending on the organism.[24] CNCs can be extracted from wood pulp, cotton, several bacteria and tunicates.[6,7] The CNCs used for the experiment here were prepared by hydrolysis of 92-Alpha eucalyptus sulphite pulp. AFM studies of the resulting CNCs (see the Supporting Information, SI) revealed average diameters of 6 ± 1.5 nm and lengths of 90 ± 40 nm. For all microscopy investigations, 3 mL of CNC suspension was placed in a 1 inch polystyrene petri dish lid, which is cleaned with water and dried under nitrogen.

In order to study the evolution of color formation while drying CNC films in a controlled and reproducible fashion, a stainless steel environmental chamber with a quartz window was used. The humidity inside this chamber was adjusted using a solvent vapour annealing setup with two independent gas lines where the nitrogen flow was controlled by a computer.[25] In one of the lines the nitrogen was bubbled through deionised water in a thermostated wash bottle at 21 °C, before combining with the dry nitrogen of the other line. The variation of the relative flow rates in the two lines adjusts the relative humidity of the output flow that was then fed into the sample chamber. The output was passed through a second water-filled wash bottle, completing the seal of the system.

The chamber was mounted on the stage of a modified BX 60 Olympus microscope equipped for polarisation spectroscopy. This setup allowed us to simultaneously collect circular polarisation-resolved spectra and microscope images of the sample during the evaporation process. The drying process was monitored at regular intervals of 10 minutes; collecting two images and two spectra with left- and right-handed circular polarisation for each time point.

We can optically distinguish three different stages of film formation. At the beginning the CNCs are isotropically suspended allowing direct imaging of the interface between air and the cellulose suspension. At this stage, by varying the focal point location inside the sample, it was confirmed that there are no other interfaces apart from the bottom water-petri dish interface. This observation shows that the CNCs do not precipitate out before drying, and remain suspended as they form into larger-scale assemblies. The reflected light was spectrally homogeneous across the visible spectrum with a reflectivity of (2 ± 0.5)%, in agreement with predictions using the Fresnel coefficients of an air-aqueous suspension interface.

The second stage sets in after partial water evaporation, resulting in the formation of a gel phase, i.e. the liquid becomes viscous. At this point, it is still possible to distinguish the gel-air and gel-petri dish interfaces and the reflectivity remains spectrally flat but increases to 3 ± 0.5%. This indicates an average refractive index of the gel of n_G_ = 1.42, allowing the cellulose concentration in the gel to be estimated using the Maxwell-Garnett approximation. From n_water_ = 1.33 and n_CNCs_ = 1.56, a CNC volume fraction of 40% is obtained, implying a concentration increase by a factor of 40. Note that tracking the focal position of the air-solution interface during drying is fundamental to measuring the correct reflectivity values. To this end we implemented an automated autofocus procedure that adjusts the position of the stage to compensate the variation of interfacial position caused by water evaporation and drift of the stage during the experiment.

During this phase, it is possible to identify domains with a stacked structure (known as tactoids).[26,27] In **Figure 2**(a) the arrows indicate the position of tactoids near the interface with air (seen as bright/dark periodic stripes). From these images it is possible to directly measure the distance between the tactoid planes, which progressively decreases as evaporation proceeds, down to 4 μm. Closer spacing values drop below the spatial resolution of the low numerical aperture objective necessary in this study.

**Figure 2:**
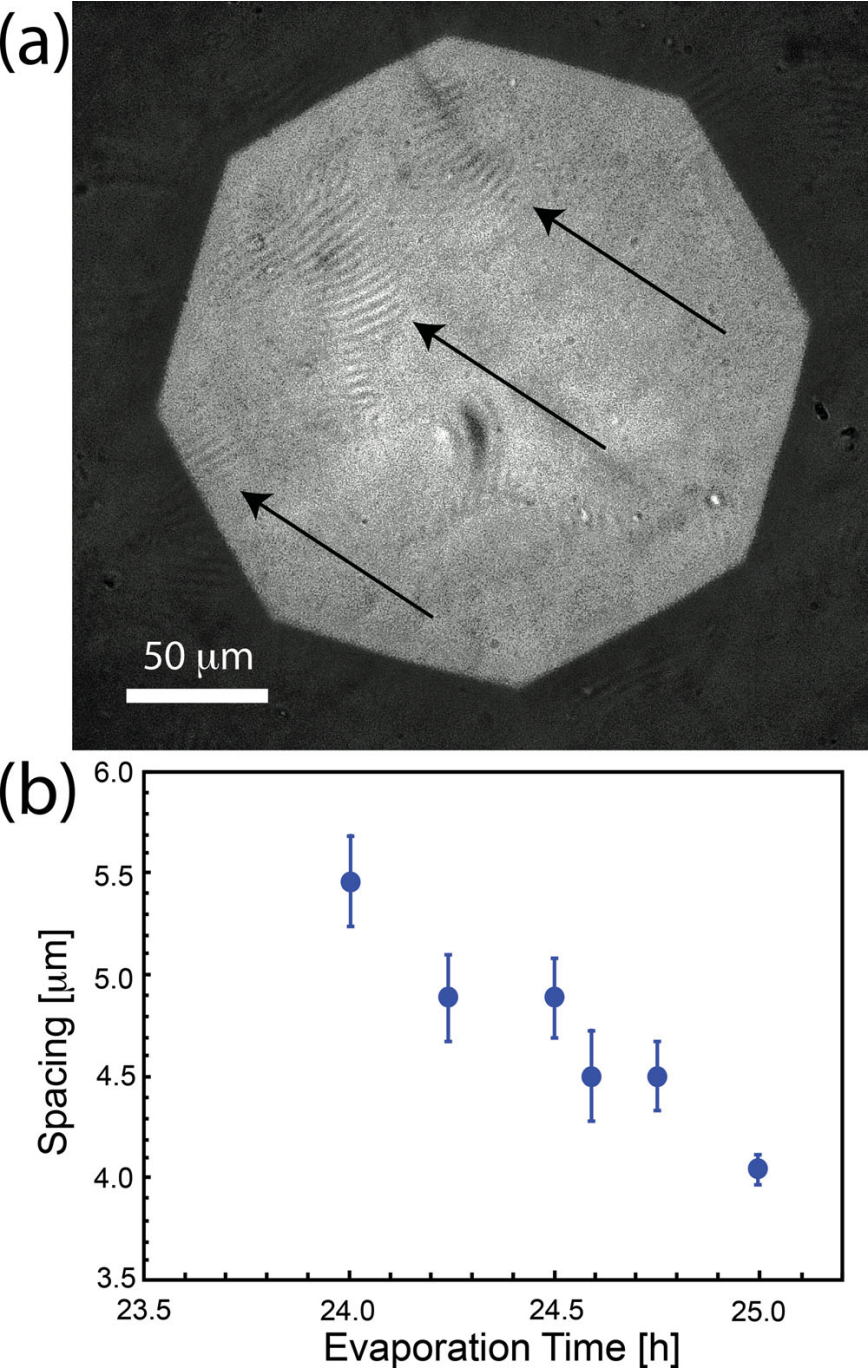
Tactoid formation at the second stage of evaporation. (a) Optical micrograph of tactoids obtained after 25 h of evaporation in atmosphere with a humidity of 80%. The arrows highlight the positions of the tactoids. (b) Inter-plane distances measured from microscopy images as a function of the evaporation time.

In the third stage, the film becomes colored but only in the left circular polarisation channel. The reflection in the opposite channel is only a few percent with the flat wavelength variation stemming from the air-film interface (see Figure SI2 of the SI). **Figures 3**(a,b) show polarization-resolved spectra obtained during the evaporation process. A near infrared reflection is visible after 23 h of evaporation at 50% humidity and the peak position then shifts from 750 nm to 460 nm. This peak shift varies non-linearly with time, as seen in Figure 3(b). A rapid change from the near infrared to 550 nm is followed by a slower color evolution during the next few hours, followed by another quick change to the final color of the film. Between these last two stages, the tactoids, which were previously randomly oriented in the suspension, align to have the nematic axis perpendicular to the petri dish surface. We speculate that the water meniscus mainly drives this process. This is supported by our observation that the order is well maintained in the centre of the sample where the evaporation sets in faster and surface tension provides a powerful alignment force. At the edge of the sample where the meniscus is not parallel to the surface of the petri dish (due to the hydrophobicity of its wall) we do not observe only ordered chiral nematic orientation.

**Figure 3:**
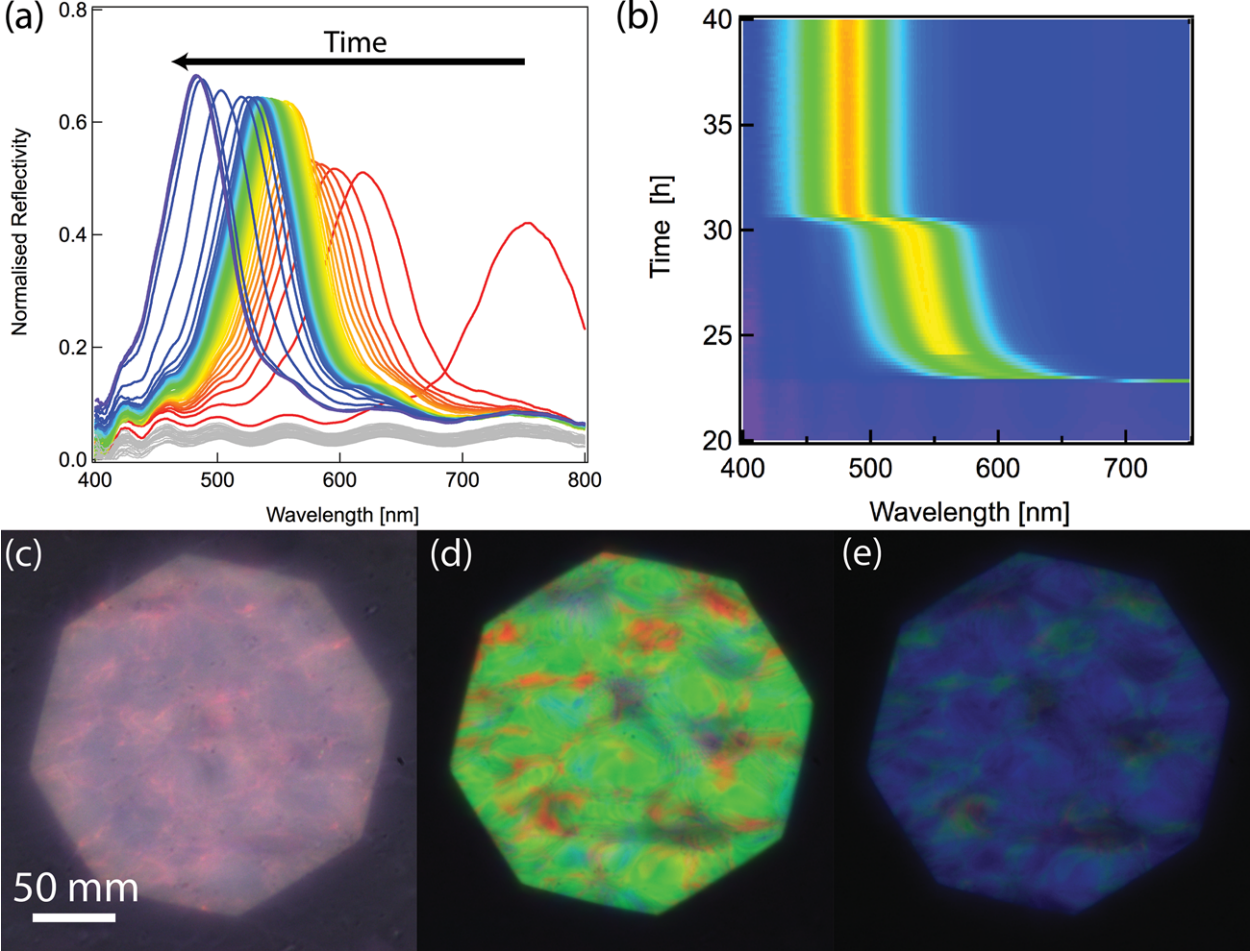
Color variation of a drying film prepared from a 1.1% suspension of cellulose CNCs. (a) Selected reflection spectra during evaporation. The red-to-blue color scale represents the evaporation time. The traces in grey are from the earlier stage of the evaporation process when the suspension has not formed its color yet. (b) Time evolution of the peak, which appears after 23 h, with reflected intensity plotted on a blue-to-red color scale. (c–e) Images collected in left circular polarisation for increasing film formation times of 23, 25, and 40 h, respectively. The contrast in (c) is low because of the limited sensitivity of the camera in the near-infrared.

The spatially averaged spectra in Figures 3(a,b) are spatially imaged in Figures 3(c–e). The colors in Figure 3(c) correspond to the spectrum with a peak position at 750 nm and while the main part of the reflection peak is in the infrared, the color is not fully homogeneous across the image and the broadband background reflection can also be observed. Once the reflection peak shifts to the visible range, bright images are observed, Figures 3(d,e). Note that color reflection was observed exclusively in the left circular polarisation channel. Although the images in Figures 3(c,d,e) are not perfectly homogeneous, the spectra in Figures 3(a,b) are collected from an area large enough to average out this effect.

The film formation time can be varied by the choice of humidity in the sample chamber. To probe the effect of faster evaporation, films are prepared under humidity of 0%, 50% and 80% from a 1.1% by weight CNC suspension. The peak wavelength during the solvent evaporation is compared for the three different humidity conditions in **Figure 4**, at a constant flow rate of 100 cm^3^ min^−1^. In a dryer atmosphere the onset of the selective reflection was at 16 h, which increased to 27 h for 80% humidity. In all three cases, the variation of reflection spectral position with time was similar: a fast initial phase followed by a plateau wavelength and a subsequent rapid descent to the final color. Interestingly, the ultimate peak wavelength depends on the evaporation conditions (see Figure 4(b)), with fast-drying films blue-shifted compared to slow-drying films, despite using the same starting CNC suspension.

**Figure 4:**
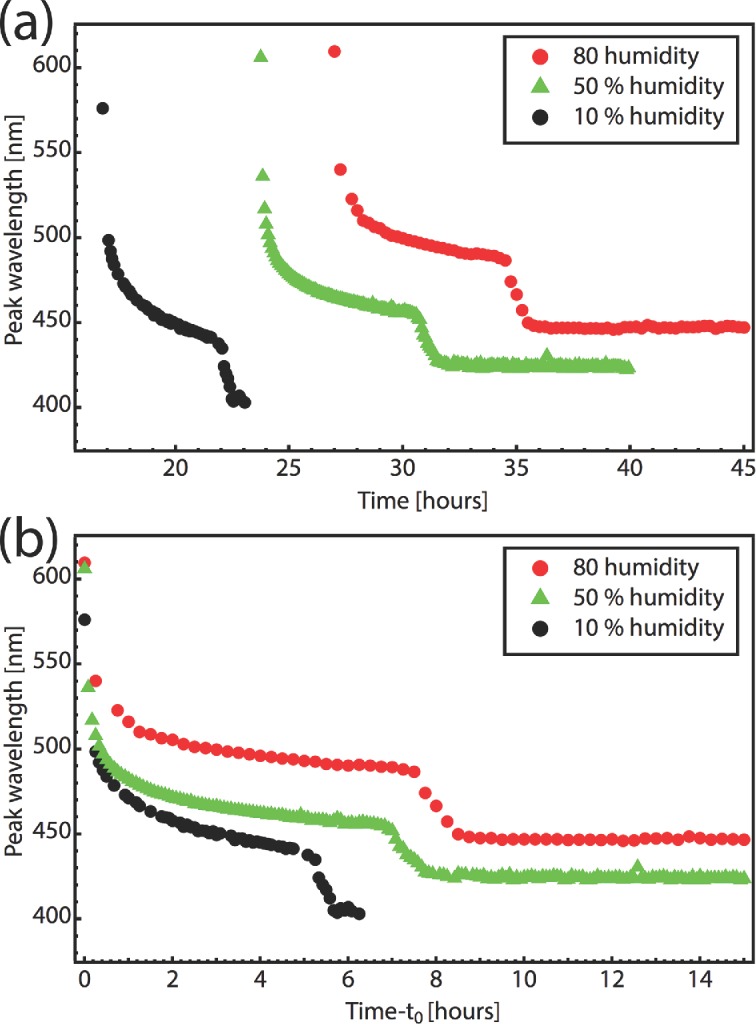
Time evolution of reflectance peak during film formation at three different humidities. (a) Peak wavelength plotted as a function of elapsed time since the start of the experiment and (b), since the reflectance peak was first detected at time t_0_.

Two effects contribute to the color change during drying. Initially, when there is still excess of water present, the reflection spectral shift is caused predominantly by a change in the pitch of the helical stack during water evaporation. This slows down as the water content reduces. A second faster change (within 30 min) then initiates suddenly after several more hours of drying. We believe that is due to the additional ejection of remaining water and a consequent increase of the refractive index contrast. This transition, which occurs always at the same CNC concentration, has not been previously reported and it is crucial to understand for predicting the final color of the assembled nanostructure stack. In this second stage of the film formation, a few percent compression occurs, but continued water evaporation does not produce a large enough shift of the peak to fit the experimental data, hence the refractive index must rise, supported by the increase in peak reflectivity also observed.

The optical properties of the films were further investigated using a goniometer setup sketched in **Figure 5**(a).[5] The optical properties of the analysed films are sufficiently uniform to probe an area of about 1 mm^2^. A collimated beam from deuterium and tungsten lamps is focused on the sample at a variable angle *θ_i_* and the reflected or scattered light is collected at *θ_d_* and focused into an optical fibre that is coupled into the spectrometer. The angular resolution for this configuration is about 5 degrees. The specular reflection as a function of *θ_i_* (Figure 5(b)) shows an 80 nm peak shift, which is responsible for the characteristic iridescent appearance of the film. A similar behaviour is predicted by calculations using the Berreman method[28]–[30] shown in Figure 5(c,d), but the width of the measured peak is significantly larger than the prediction. Since this is presumably due to inhomogeneities in the film, the scattering properties of the samples were also investigated, (Figure 5(e,f)). The peak signal decreases by about one order of magnitude for angles deviating more than 3 degrees from the specular reflection peak, indicating a good alignment of the chiral nematic axis with respect to the surface over the detected sample area. The larger peak width is therefore probably associated with the non-homogeneous distribution of inter-layer pitch in the investigated area. From the calculation we observe that a 10% variation in pitch would account for the linewidth observed, which is also well supported by the images in Figure 3. We thus confirm that CNCs in solution progressively assemble into helical layer stacks that compress as water is extracted, assembling into ordered chiral films on top of the substrate.

**Figure 5:**
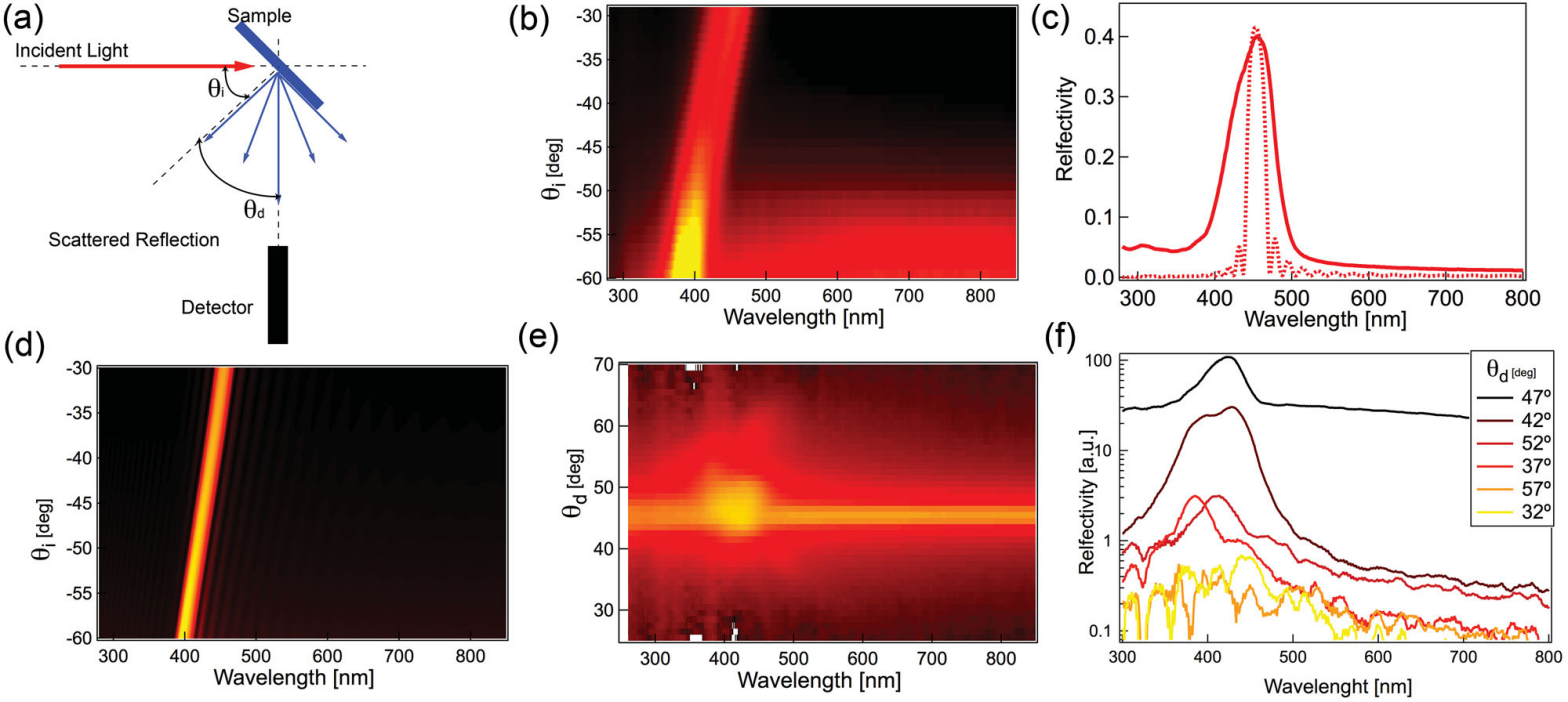
(a) Scattering setup, with collimated light focused onto the sample, while angles of incidence *θ_i_* and detection *θ_d_* can be independently varied. (b) Spectral distribution of specular light reflection (*θ_i_* = *θ_d_*) as a function of *θ_i_*. (c) Comparison between measured (continuous line) and simulated (dotted line) reflection spectra for an angle of incidence *θ_i_* = 30°. (d) Simulated specular reflection spectra for the same parameters as in (b). (e) Scattering measurement for a fixed *θ_i_* = 45°. (f) Scattering spectra for several detection angles normalised with respect to a white Lambertian diffuser.

In conclusion, we monitored in detail the self-assembly process of colored biomimetic cellulose films. By investigating the spectroscopy of drying film in controlled humidity conditions, we are able to tune the optical properties and distinguish three different phases of cellulose organisation into a chiral nematic order. In particular, it is possible to fine-tune the color of the self-assembled film by carefully adjusting the evaporation conditions. Detailed scattering studies and comparison with theoretical predictions allow the quantification of disorder within the sample. These indicate that the main cause of the color fluctuations in such films is due to a non-uniform helical pitch, and not to the misalignment of the chiral nematic director. These studies pave the way to create sustainable colored materials, as well as the use of cellulose-based optics for edible bio-sensors.

## Experimental Section

*CNC Preparation*: To prepare the CNCs the wood pulp was dissolved in 64 w/w% sulphuric acid at 45 °C under continuous stirring for 1 h (ratio of cellulose to acid is kept at 1:9). The cellulose–sulphuric acid solution was then diluted 10 times with cold deionised (DI) water to stop the hydrolysis reaction and allowed to settle overnight. The clear top layer was removed and the remaining white suspension was centrifuged. After centrifugation, the supernatant was removed and the resulting thick white suspension was first diluted with DI water and re-concentrated with centrifuge 3 more times to remove all soluble cellulosic materials. The white suspension obtained after the last centrifugation step was then dialysed. We use dialysis membrane tubes with a 12 000–14 000 molecular weight cut-off (Spectrumlabs/Spectrapor membranes) and dialysed against slow running pure water for 2–4 weeks or until the CNCs suspension reached a pH of 6. The final step was the concentration of CNC suspension of 1.1 w/w% by centrifugation.

*Optical Measurements*: Evaporation of the films was monitored using an optical microscope equipped for spectroscopy. Using a long working distance objective (20× Nikon, NA = 0.35, WD = 22 mm), unpolarised light from a halogen lamp was focused into the sample located inside the chamber. Light reflected from the sample was then collected through the same objective (in bright-field configuration) and filtered with a super-achromatic quarter-wave plate combined with a polariser. Rotating the quarter-wave plate at a fixed position with respect to the linear polariser, left- or right-handed circularly polarised light was selected. A 50–50 beam splitter divided the detected light into a color-CCD camera and a spectrometer (Ocean Optics USB65000), using a 600 μm-core optical fibre in confocal configuration.[2,5] For additional details on the goniometer setup used during the experiment please refer to reference.[5]
