# Supplementary material for: Controlled, Bio-inspired Self-Assembly of Cellulose-Based Chiral Reflectors
Source: Adv Opt Mater. 2014 May 30;2(7):646–50. doi: 10.1002/adom.201400112 (PMC4515966; doi:10.1002/adom.201400112)
Supplement: Supplementary file 1 [file adom0002-0646-sd1.pdf]

Copyright WILEY-VCH Verlag GmbH & Co. KGaA, 69469 Weinheim, Germany, 2014.

ADVANCED OPTICAL MATERIALS

Supporting Information

for *Advanced Materials.,* DOI: 10.1002/adom.201400112

Controlled, Bio-inspired Self-Assembly of Cellulose-Based Chiral Reflectors

*Ahu Gumrah Dumanli, Gen Kamita, Jasper Landman, Hanne van der Kooij, Beverley J. Glover, Jeremy J. Baumberg, Ullrich Steiner, and Silvia Vignolini**

Copyright WILEY-VCH Verlag GmbH & Co. KGaA, 69469 Weinheim, Germany, 2013.

Supporting Information

Controlled bio-inspired self-assembly of cellulose-based chiral reflectors

Ahu Gumrah Dumanli[12], Gen Kamita[1], Jasper Landman[1], Hanne van der Kooij[1], Beverley. J. Glover[1], Jeremy J. Baumberg[1], Ullrich Steiner[1], Silvia Vignolini[1,2,]*

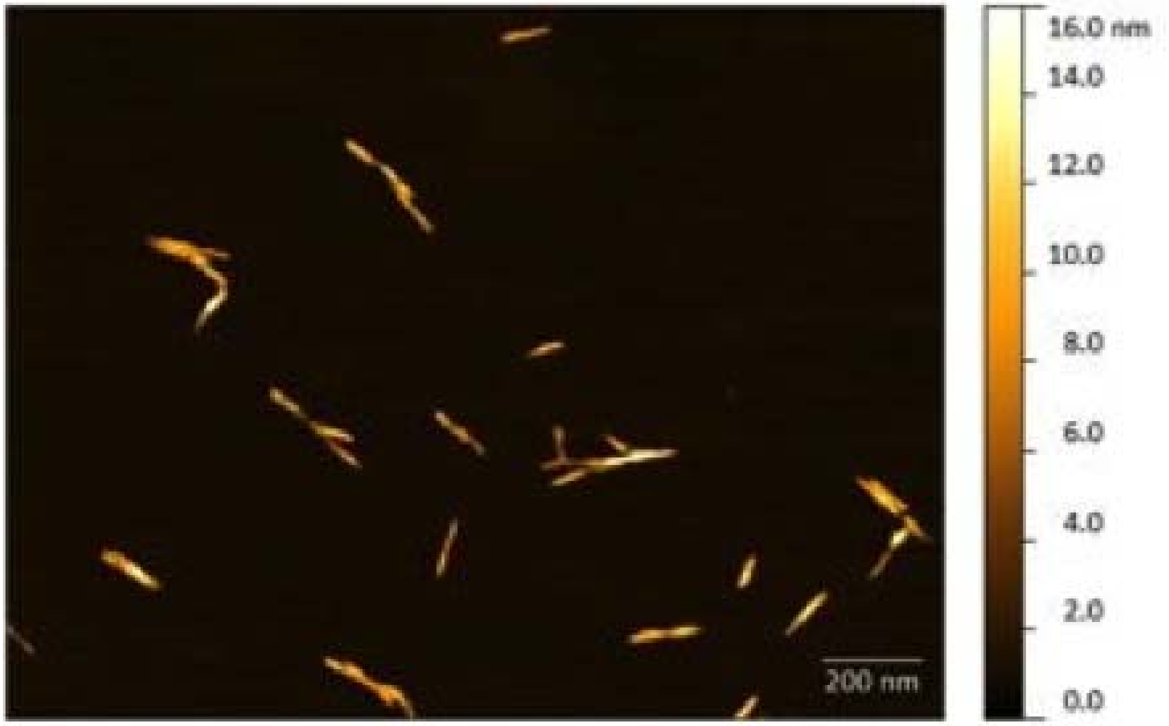

Figure S1. AFM image of the CNCs used in this study in height profile mode, cast onto a freshly cleaved mica surface. The length of the nanocrystals was estimated as the log-normal average of the length of the long axis of 291 non-aggregated nanocrystals. The diameter of the nanocrystals was estimated as the normal average of the height of the centre of 114 non-aggregated nanocrystals. These heights were determined from line profiles transverse to the long axis of the NCCs.

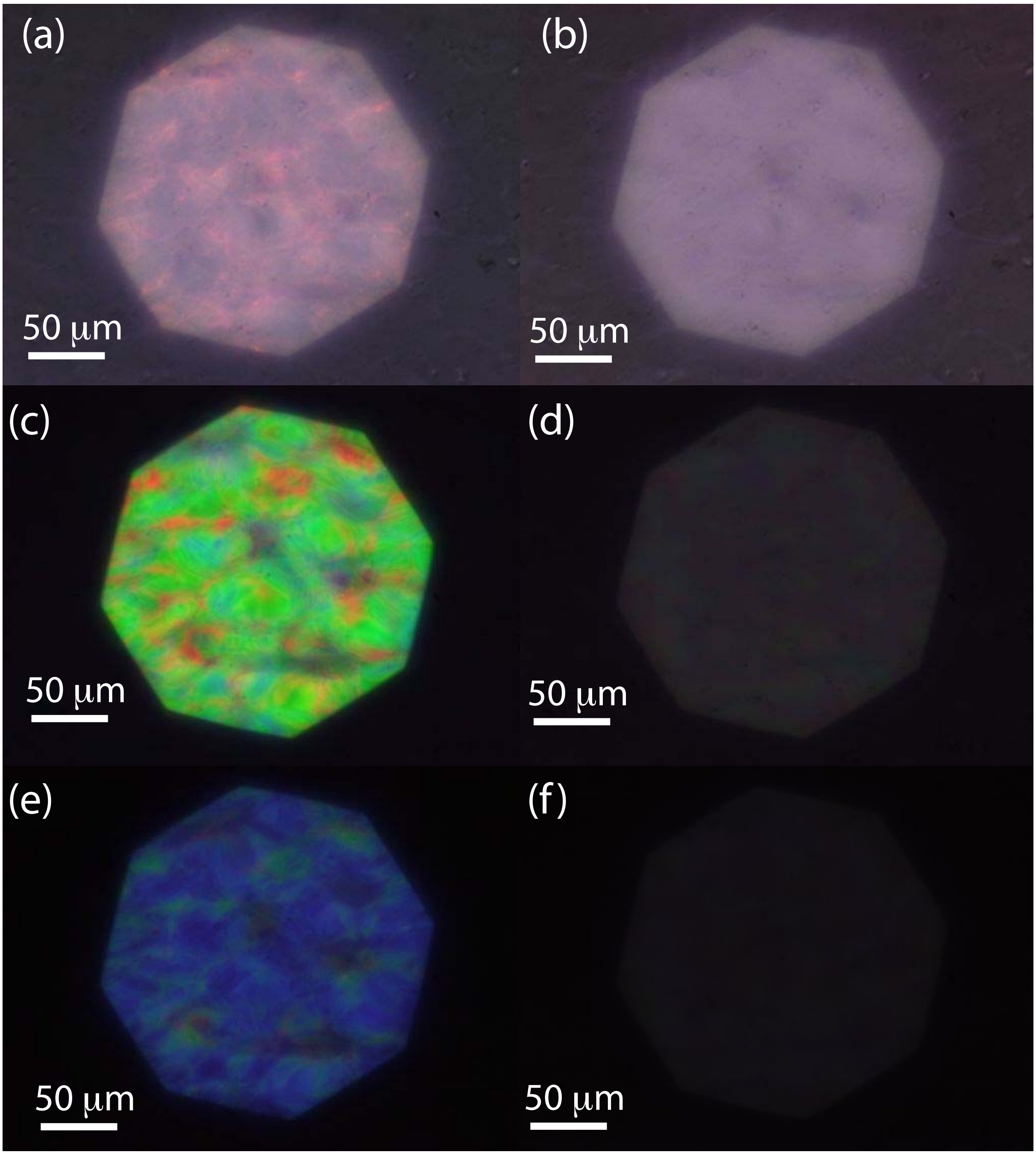

Figure S2. Colour variation of a drying film prepared from a 1.1 % suspension of cellulose CNCs in the Left and Right circular polarisation channel. The sample was exposed to a flow of 100cm^{3}min^{1-} of air with a humidity of 50%. Images collected in the left circular polarisation channel for increasing film formation times of 23 **(a,b),** 25 **(c,d)** and 40 hours **(d,e)**,in the circular left **(a,c,e)** and right **(b,d,f)** polarisation channel. Integration time in the camera is decreased to avoid saturation, see spectra of **Figure 3(a)** in the manuscript.

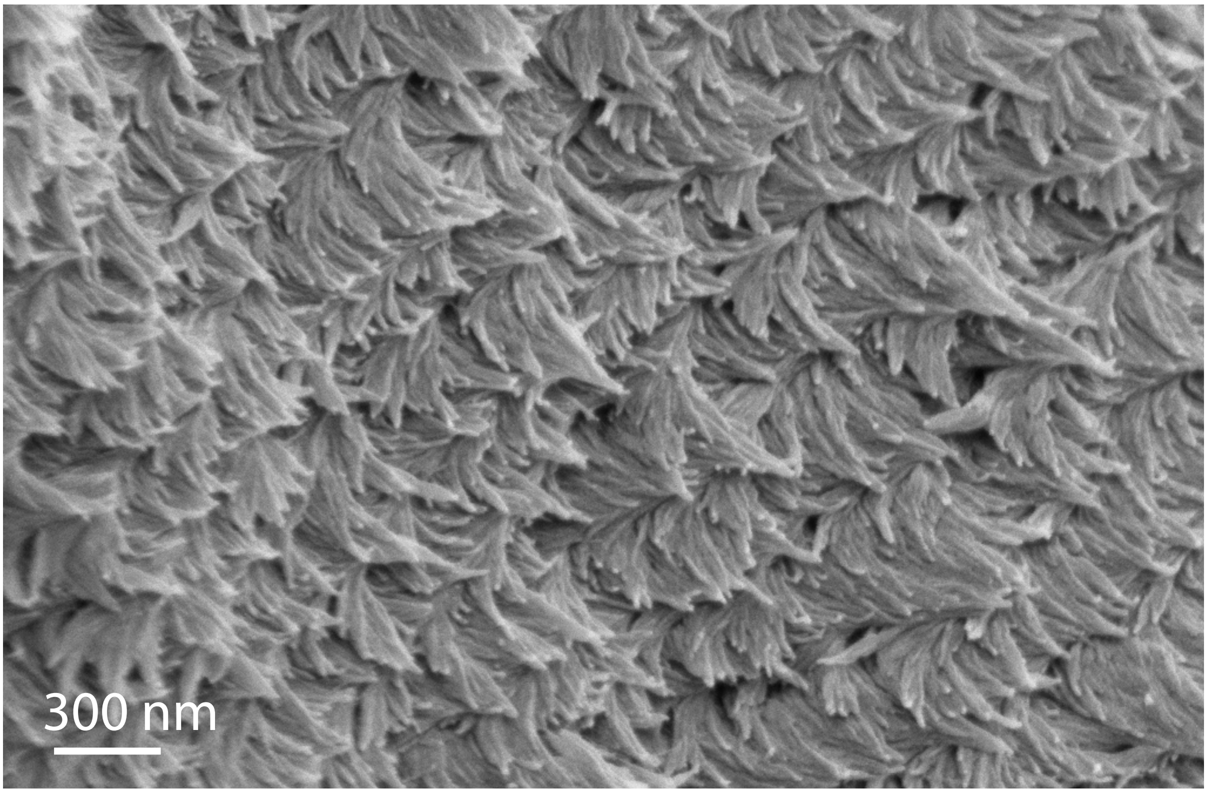

Figure S3. Scanning Electron microscope image of the cross section of the cellulose film in shown in **Figure 1(a)**.

Movie M1: Each frame of the movie corresponds to an image collected using an optical microscope (as explained in the experimental section) during the evaporation process.
